# Comparison of multi-delay FAIR and pCASL labeling approaches for renal perfusion quantification at 3T MRI

**DOI:** 10.1007/s10334-019-00806-7

**Published:** 2019-12-06

**Authors:** Anita A. Harteveld, Anneloes de Boer, Suzanne Lisa Franklin, Tim Leiner, Marijn van Stralen, Clemens Bos

**Affiliations:** 1 Department of Radiology, University Medical Center Utrecht, Utrecht University, Postbox 85500, 3508 GA Utrecht, The Netherlands; 2 Department of Radiology, C.J. Gorter Center for High Field MRI, Leiden University Medical Center, Leiden, The Netherlands

**Keywords:** Arterial spin labeling (ASL), Arterial transit time, Magnetic resonance imaging, Kidney, Renal blood flow

## Abstract

**Objective:**

To compare the most commonly used labeling approaches, flow-sensitive alternating inversion recovery (FAIR) and pseudocontinuous arterial spin labeling (pCASL), for renal perfusion measurement using arterial spin labeling (ASL) MRI.

**Methods:**

Multi-delay FAIR and pCASL were performed in 16 middle-aged healthy volunteers on two different occasions at 3T. Relative perfusion-weighted signal (PWS), temporal SNR (tSNR), renal blood flow (RBF), and arterial transit time (ATT) were calculated for the cortex and medulla in both kidneys. Bland–Altman plots, intra-class correlation coefficient, and within-subject coefficient of variation were used to assess reliability and agreement between measurements.

**Results:**

For the first visit, RBF was 362 ± 57 and 140 ± 47 mL/min/100 g, and ATT was 0.47 ± 0.13 and 0.70 ± 0.10 s in cortex and medulla, respectively, using FAIR; RBF was 201 ± 72 and 84 ± 27 mL/min/100 g, and ATT was 0.71 ± 0.25 and 0.86 ± 0.12 s in cortex and medulla, respectively, using pCASL. For both labeling approaches, RBF and ATT values were not significantly different between visits. Overall, FAIR showed higher PWS and tSNR. Moreover, repeatability of perfusion parameters was better using FAIR.

**Discussion:**

This study showed that compared to (balanced) pCASL, FAIR perfusion values were significantly higher and more comparable between visits.

**Electronic supplementary material:**

The online version of this article (10.1007/s10334-019-00806-7) contains supplementary material, which is available to authorized users.

## Introduction

Renal perfusion is a valuable physiological parameter for assessing kidney function and identifying pathology [1]. In recent years, arterial spin labeling (ASL) magnetic resonance imaging (MRI) has been emerging as a method for measurement of renal perfusion [2] that does not warrant administration of contrast agent [3–5] or the involvement of lengthy invasive clearance measurements [6, 7]. An additional advantage of ASL is that it enables measurement of local perfusion, in contrast to clearance techniques which only assess renal blood flow of both kidneys combined. In ASL, images are acquired with (label) and without (control) inverting/saturating magnetization of arterial blood flowing into the tissue. The signal intensity present in the subtracted label–control images is proportional to perfusion of the tissue [8].

Renal perfusion is a relatively new application area of ASL-MRI. In the white paper for brain ASL [9], pseudo-continuous ASL (pCASL) is the recommended labeling approach. However, the most commonly used labeling approach for renal ASL thus far has been flow-sensitive alternating inversion-recovery pulsed ASL (FAIR) [2]. In general, FAIR has the advantage of higher labeling efficiency and lower specific absorption rate, whereas pCASL can achieve a higher intrinsic signal-to-noise ratio (SNR) [9, 10].

ASL-MRI in the abdomen brings new challenges with respect to the brain, like respiratory motion, complex vasculature, and increased magnetic field inhomogeneities due to the proximity of air in the lungs and in the digestive tract. Thus far, no studies have been performed directly comparing different labeling approaches for renal ASL, and therefore, the effect on the obtained perfusion signal remains unclear. The aim of this study was to compare ASL-MRI of the kidneys with the two most commonly used labeling approaches FAIR and pCASL, to obtain a better insight into the reliability and repeatability of each method and to identify the most efficient method to perform renal perfusion measurements.

## Methods

### Study population

Between March and November 2018, volunteers aged > 40 years, without history of renal disease or contraindications for MR imaging, were included. This prospective study was approved by the local institutional review board and all subjects provided written informed consent. The scans used in this study were acquired as part of the multiparametric repeatability ReMaRK study (Repeatability of functional Magnetic Resonance imaging of the Kidneys).

### MR imaging

Imaging was performed on a 3T MR system (Ingenia, Philips, Best, The Netherlands; release 5.3.1) using a body coil for transmission and a 28-element phased array coil for reception. All subjects were scanned twice with an interval of 1 week between acquisitions (median 7 days; range 4–14 days). To control gastrointestinal physiological conditions, both visits were scheduled at the same time of the day (afternoon), and subjects were asked to drink 2 L of non-alcoholic liquids and to avoid salt and protein rich meals prior to the MRI examination. Prior to the first-scan session, blood was sampled from each subject to determine kidney function by calculating the eGFR with the CKD-EPI formula (Chronic Kidney Disease EPIdemiology collaboration [11]) using the measured creatinine.

#### Scan protocol

The scan protocol consisted of both FAIR and pCASL perfusion imaging, both with varying delay times between labeling and readout, and auxiliary sequences to estimate equilibrium magnetization (M_0_) and T_1_ for perfusion quantification.

FAIR labeling was implemented as previously described [12]. In this method, label and control conditions are achieved by alternating between a selective and non-selective inversion slab. An adiabatic FOCI (frequency offset-corrected inversion) pulse was used for the selective inversion [13, 14]. Pre-saturation using WET [15] (water suppression enhanced through T_1_ effects) consisting of four pulses and post-saturation using a single 90° pulse were applied to the imaging region directly before and after each selective or non-selective inversion pulse, respectively, to minimize perfusion-weighted signal differences caused by inversion efficiency differences between both inversion slabs. The selective inversion slab was aligned with the imaging stack and was 10 mm wider (thickness 54 mm) than this stack (thickness 34 mm). To define the temporal bolus width of the labeled spins, a QUIPSS II scheme was applied at a specific delay time (TI_1_) after the inversion pulse (five saturation pulses timed equidistantly within 100 ms after TI_1_) that was placed anterior of the imaging stack with a gap of 10 mm with the aim to cover the feeding arteries, for the kidneys specifically the descending aorta [16, 17]. The thickness of this saturation slab was 120 mm. FAIR data were acquired with four different times-to-inversion (TI; 0.8, 1.4, 2.0, and 2.6 s) and QUIPSS II at TI_1_ of 1.2 s.

pCASL labeling was performed using the balanced version [18], as implemented by the vendor. In this method, flowing spins are inverted by a long train of short, repeated RF pulses (Hanning-shaped, duration 480 μs, spacing 1210 μs, average B_1_ 1.5 μT) in combination with a switching selection gradient (average strength 0.36 mT/m) to achieve the label condition. WET water suppression was applied to the imaging region directly before the labeling, to eliminate residual magnetization modulation from the previous acquisition. pCASL data were acquired with four different post-labeling delays (PLD; 0.5, 1.0, 1.5, and 2.0 s) and a label duration of 1.5 s.

Each delay time was obtained in a separate acquisition that consisted of ten label–control pair repetitions. The M_0_ scan, essentially the FAIR/pCASL scan without labeling or suppression pulses, was acquired four times to improve the SNR. The T_1_ map was acquired using a cycled multislice inversion-recovery sequence [19] with 11 inversion times (range 55–2035 ms). All scans were performed with the same single-shot gradient echo EPI 2D multislice readout. Detailed scan parameters are provided in Table 1.

**Table 1:** Scan parameters

Parameters	ASL	M_0_	T_1_ map
TR/TE (ms)	6500/22	6500/22	6500/22
EPI factor	65	65	65
Flip angle (°)	90	90	90
SENSE	1.5 (FH direction)	1.5 (FH direction)	1.5 (FH direction)
FOV (mm^3^)	244 × 244 × 34	244 × 244 × 48	244 × 244 × 76
Acquired voxel size (mm^2^)	3 × 3	3 × 3	3 × 3
Slice thickness (mm)	6	6	6
Slice gap (mm)	1	1	1
No. of slices	FAIR: 5 pCASL: 7	7	11
Phase encoding direction	FH	FH	FH
Fold-over suppression	Saturation slabs^a^	Saturation slabs^a^	Saturation slabs^a^
Fat suppression	SPIR	SPIR	SPIR
Slice orientation	Coronal	Coronal	Coronal
Slice scan order	AP	AP	AP
No. of repetitions	10^b^	4	N/A
Delay time (s)	FAIR^c^: 0.8, 1.4, 2.0, 2.6 pCASL: 0.5, 1.0, 1.5, 2.0	N/A	N/A
Inversion time (ms)	N/A	N/A	55–2035; steps 198
Total acquisition time (min:s)	02:23	00:32.5	01:11.5

*ASL* arterial spin labeling, *TR* repetition time, *TE* echo time, *EPI* echo planar imaging, *SENSE* sensitivity encoding, *FOV* field-of-view, *SPIR* spectral pre-saturation with inversion recovery, *FH* feet–head, *AP* anterior–posterior

^a^Spatial saturation slabs superior and inferior to the image volume to suppress undesired signal aliasing

^b^Label–control pairs per delay time

^c^QUIPSS II saturation pulses were set at TI_1_ = 1.2 s; delay time 0.8 s was, therefore, obtained without these saturation pulses

A localizer scan was acquired to enable correct planning of all subsequent scans. The image readout of all scans was planned coronal oblique along the long axis of the kidneys to minimize through plane motion of the kidneys; the center slice had identical geometry for all scans. For FAIR scans, care was taken to exclude the feeding arteries of the kidneys, especially the descending aorta, from the selective inversion slab and to include them within the QUIPSS II slab. For pCASL scans, the same procedure was applied during each scan session to plan the labeling slab. The labeling slab was placed perpendicular to the descending aorta, approximately 10–11 cm above the center of the kidneys. Care was taken that the labeling slab was placed above the kidneys to prevent the kidneys from sliding into the labeling slab during breathing, but below the diaphragm to minimize susceptibility artifacts from the air–tissue interface at the lungs. The planning of both labeling approaches is shown in Supplementary Figure S1. B_0_ shimming was applied to the imaging stack and the labeling slab independently. B_1_ shimming was applied to a volume shim box that covered the entire field-of-view. All acquisitions were performed with paced breathing. Subjects were instructed before scanning and coached during scanning to synchronize breathing to the TR of 6500 ms of the sequence and to briefly hold their breath in expiration during the image readout [20]. Labeling was timed directly after expiration. A bellows was placed on the subject’s upper abdomen to monitor respiration and the compliance with the breathing instructions.

### Image processing

**Fig. 1:**
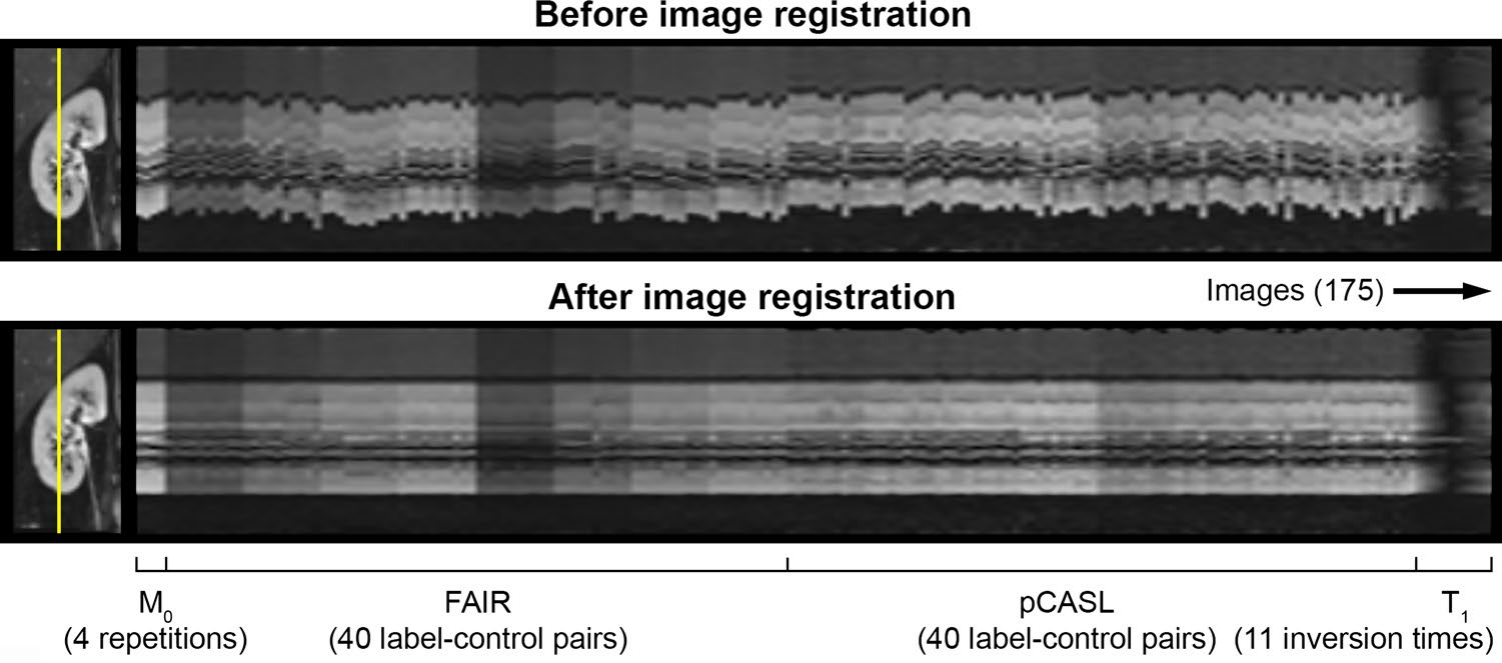
Example images of the right kidney before and after image registration. Improved alignment can be observed for the images after motion correction. Images visualize the same intersection (yellow line in the coronal image) over all M_0_, ASL, and T_1_ images

Image processing and analysis were performed using custom scripts in MeVisLab (version 2.8.2; MeVis Medical Solutions AG, Bremen, Germany). Processing was performed per subject on images obtained during the same visit, separately for each kidney. After a wide crop around each kidney was made, slice-wise motion correction was performed using a groupwise image registration method [21] implemented in Elastix [22] to compensate for misalignments due to (respiratory) motion. ASL, M_0_, and T_1_ images were registered simultaneously. To this end, 3D slice stacks were created for each slice of the cropped image, with the non-subtracted ASL (multi-delay FAIR and pCASL), M_0_ and T_1_ slices in the third dimension, with a total of 175 images per registration; see Fig. 1. The groupwise registration method does not require the choice of a reference image, thus avoiding registration bias, and the method is robust against intensity changes between the images.

After image registration, ASL label and control images were pair-wise subtracted (ΔM). Next, for each ASL scan (delay time), outlier rejection was performed by excluding subtraction images containing > 20% voxels (within the kidney region) with a value of more than ± 2 SD from the mean voxel value over all repetitions. The remaining subtraction images were averaged per delay time to obtain the perfusion-weighted images ($\overline{\Delta M}$). Voxel-wise T_1_ relaxation times were calculated by a nonlinear least-squares fit of the 11 inversion-recovery magnitude images to a monoexponential recovery function. The M_0_ images were averaged over the four repetitions. The whole kidney region that was used for the outlier rejection was manually drawn along the visible kidney contour on the averaged M_0_ images after image registration.

Voxel-wise perfusion quantification was performed using Buxton’s general kinetic model for pulsed and continuous labeling ASL signal to estimate renal blood flow (RBF) and arterial transit time (ATT) [23]. The measured $\overline{\Delta M}$, *M*_0_, and *T*_1,tissue_ values were used as input for the two-compartment model together with assumptions for *T*_1,blood_ of 1.65 s [24], tissue–blood partition coefficient of 0.9 mL/g [25], and labeling efficiency of 95% for FAIR and 85% for pCASL. In the model fit, the exact delay time of each slice was used by taking into account the delay between slices of 65 ms.

The number of slices for the FAIR acquisition was limited by the available space between the slice-selective inversion slab and the aorta. Therefore, only five slices were processed, analyzed, and compared for FAIR and pCASL.

### Image analysis

For quantitative comparison of both labeling approaches, the following metrics were used: (1) relative perfusion-weighted signal (PWS), (2) temporal SNR (tSNR), (3) RBF, and (4) ATT. PWS was calculated for each delay time as $\overline{\Delta M}$/*M*_0_$\times$ 100%. tSNR was defined as ${\mu }_{\Delta M}/{\upsigma }_{\Delta M}$ where ${\mu }_{\Delta M}$ is the temporal voxel-wise mean and ${\upsigma }_{\Delta M}$ is the temporal voxel-wise standard deviation of the subtraction (Δ*M*) images. tSNR was also calculated for each delay time separately. The tSNR was used as a metric for consistency of the perfusion signal over repetitions. For separate analysis of the cortex and medulla, semi-automatic segmentation based on the intensity histogram of the T_1_ map was performed by a single observer to determine thresholds separating both regions. Because of the groupwise registration of FAIR and pCASL, the same segmentations were used for both analyses. When necessary, segmentations were manually adjusted with in-plane affine transformations.

### Statistical analysis

For the quantitative metrics (PWS, tSNR, RBF, and ATT), mean and SD values were calculated over all voxels inside the segmented kidney regions (cortex and medulla) for each subject. Inter-visit reliability and agreement between measurements (i.e., repeatability) of RBF and ATT were evaluated using the intra-class correlation coefficient (ICC), within-subject coefficient of variation (CV_ws_), and Bland–Altman analysis. The CV_ws_ was calculated as (SD/mean) $\times$ 100% with SD = $\sqrt{(\sum {({x}_{1}-{x}_{2})}^{2})/2n}$, where *x*_1_ and *x*_2_ are the measurement values at both visits in the same subject, *n* is the number of subjects, and the *mean* is the average over all measurements. Differences in RBF and ATT between visits, between labeling approaches, and between left and right kidney, were tested with a Wilcoxon matched-pair signed-rank test. Spearman’s rho was used to determine association of perfusion values (RBF and ATT) between both kidneys, and between RBF and eGFR. Statistical analyses were performed using R [26] version 3.6.1. A *P* value of < 0.05 was considered to be statistically significant.

## Results

### Study population

Sixteen middle-aged volunteers (eight male; age 51 ± 10 years) were included. All subjects had an eGFR in the normal range (86 ± 15 mL/min/1.73m^2^). Five data sets from four subjects were excluded from analysis due to: (1) absence of one of the labeling methods (one subject, both visits), (2) presence of severe motion differences between FAIR and pCASL (two subjects, second visit), or (3) MRI exam was not performed (one subject, second visit). This resulted in 15 complete data sets (including both FAIR and pCASL) from the first visit and 12 available complete data sets in the same subjects from the second visit that were used for analysis.

### Image processing and analysis

**Fig. 2:**
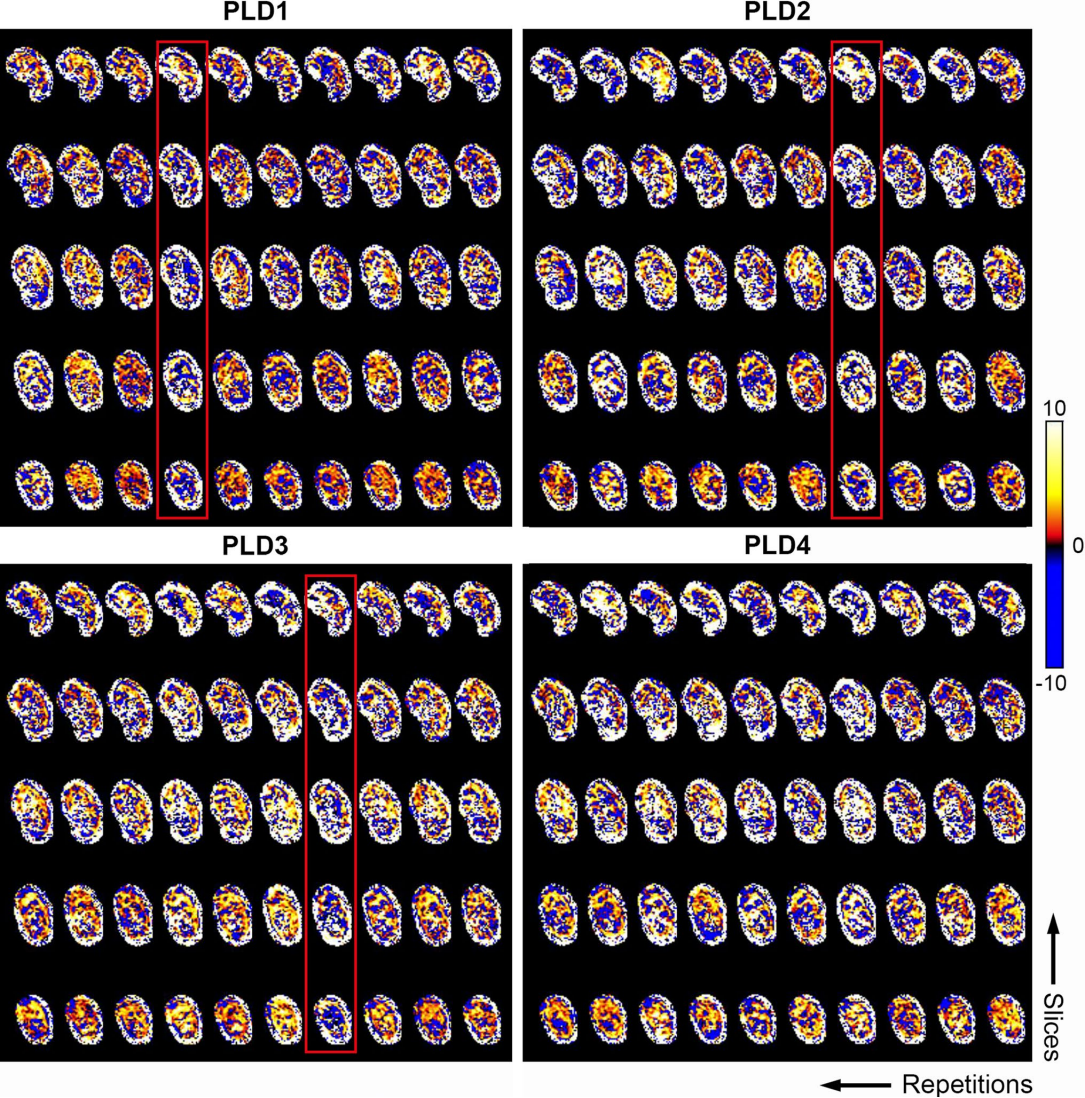
Example of outlier rejection to exclude subtraction images. Images show masked subtraction images of the left kidney with pCASL labeling before outlier rejection. Subtraction images containing > 20% voxels with a value of more than ± 2 SD from the mean voxel value over all repetitions were rejected. The label–control pairs (repetitions) that were removed after outlier rejection are highlighted in red. The color bar indicates PWS [%]. A similar example with FAIR labeling in the same subject during the same visit is shown in the Supplementary Figure S2

**Fig. 3:**
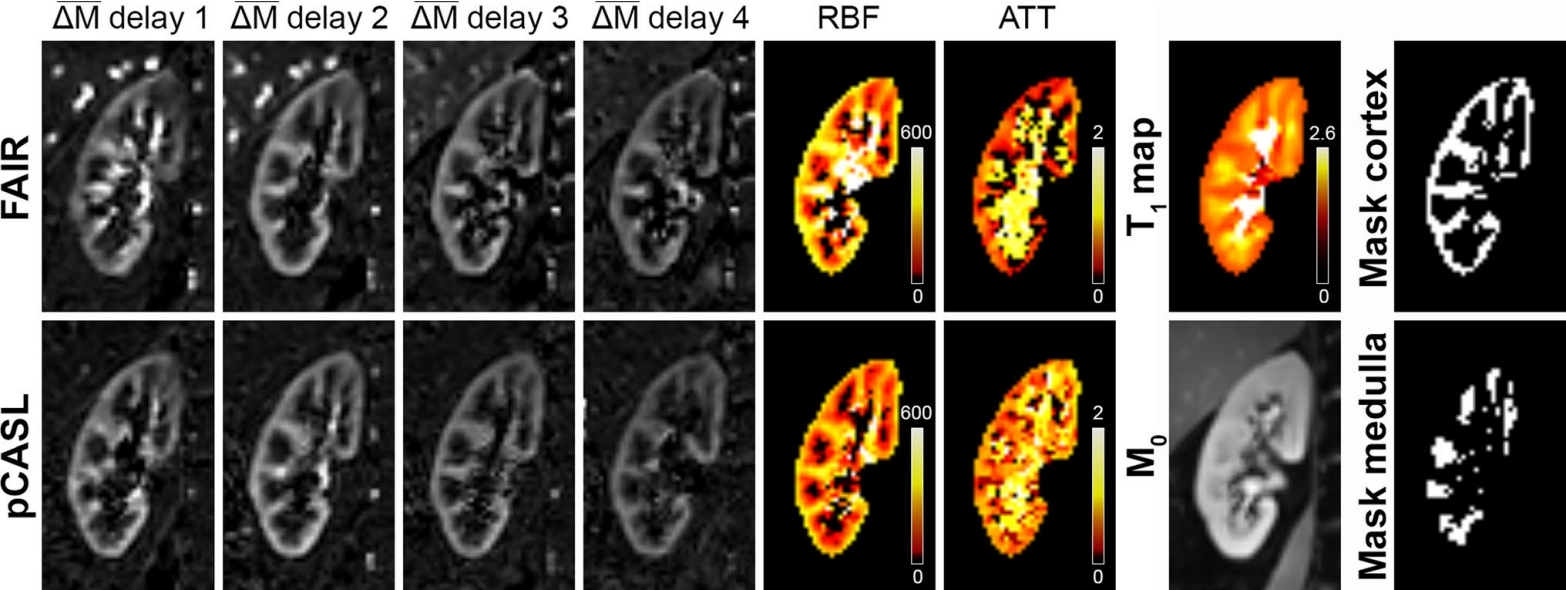
Example of processed images of the right kidney from a 41-year-old female healthy volunteer acquired at the first visit. After alignment of all scans, the multi-delay perfusion-weighted images ($\overline{\Delta M}$), *T*_1_ map (*T*_1_-relaxation time in s), and M_0_ scan were used to quantify RBF (in mL/min/100 g) and ATT (in s). Both labeling approaches were obtained with four different delay times (FAIR: 0.8, 1.4, 2.0, 2.6 s; pCASL: 0.5, 1.0, 1.5, 2.0 s). *ATT* arterial transit time, *RBF* renal blood flow

Improved image alignment after motion correction was observed in all subjects (Fig. 1). Outlier rejection was performed in 18/27 FAIR and 19/27 pCASL data sets, with a maximum of two excluded label–control pairs per delay time (Fig. 2). Manual adjustment of segmentations was necessary for 7/54 data sets of the left (*n* = 4) and right (*n* = 3) kidney. Example images after image processing and analysis of the obtained scans are shown in Fig. 3, demonstrating a clear PWS and RBF contrast between renal cortex and medulla for both labeling techniques.

### Quantitative metrics

#### PWS and tSNR

**Fig. 4:**
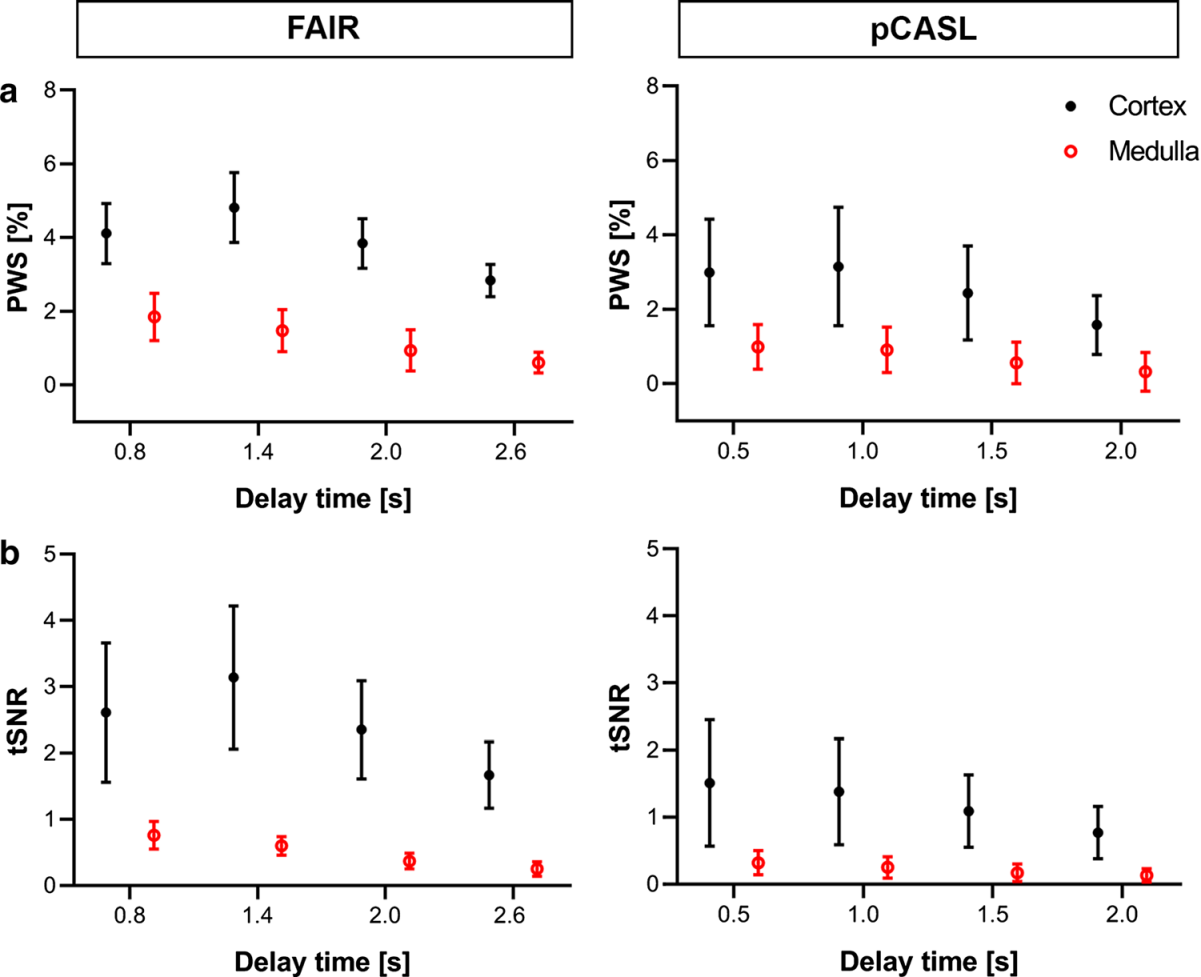
PWS (**a**) and tSNR (**b**) values obtained with FAIR and pCASL labeling approaches in the cortex (black) and medulla (red) averaged over 15 subjects at visit 1 using four different delay times. Error bars represent the standard deviation. *PWS *perfusion-weighted signal, *tSNR *temporal signal-to-noise ratio

PWS and tSNR values obtained with both labeling approaches are shown in Fig. 4. For both labeling approaches, cortical PWS showed an initial increase from the first to the second delay time and a decrease after the second delay time. For pCASL, the inter-subject variability in the PWS was much higher than for FAIR. The medullary PWS only decreased with increasing delay time. The tSNR showed similar behavior as the PWS, consistent with similar noise levels for each delay time. Although evaluated at different delay times, cortical and medullary PWS and tSNR were in general higher with FAIR, at least 37%.

#### RBF

**Fig. 5:**
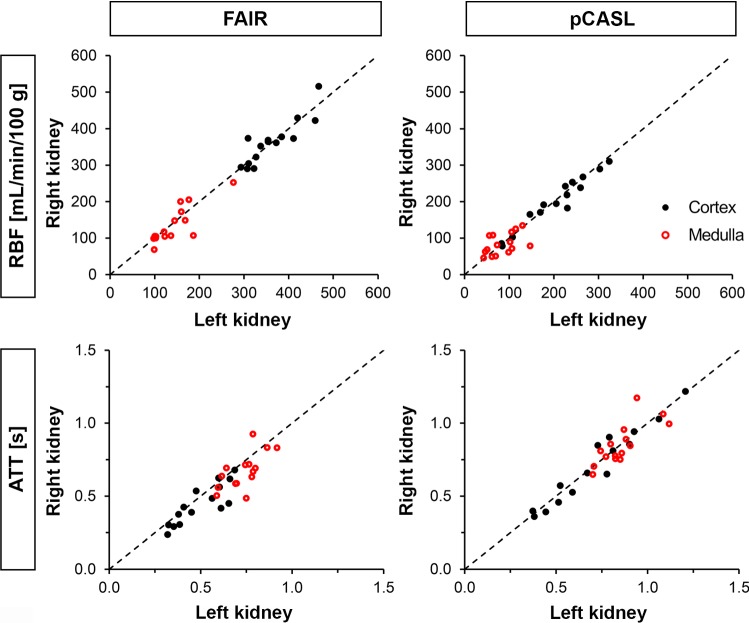
Scatterplots showing RBF and ATT values in the left and right kidney obtained with FAIR and pCASL labeling approaches in the cortex (black) and medulla (red) from 15 subjects at visit 1. *ATT *arterial transit time, *RBF* renal blood flow

Mean cortical RBF was 362 ± 57 mL/min/100 g with FAIR and 201 ± 72 mL/min/100 g with pCASL at visit 1 (*P* < 0.001). Mean medullary RBF was 140 ± 47 mL/min/100 g with FAIR and 84 ± 27 mL/min/100 g with pCASL at visit 1 (*P* < 0.001). For both FAIR and pCASL, RBF values were significantly different neither between visits (*P* ≥ 0.34; Table 2), nor between left and right kidney of a subject (*P* = 0.93 and *P* = 0.52 in cortex; *P* = 0.42 and *P* = 0.98 in medulla; for FAIR and pCASL, respectively). There was a significant correlation between the measured RBF values in the left and right kidney of each subject, both with FAIR (*r* = 0.83, *P* < 0.001 in cortex and *r* = 0.86, *P* < 0.001 in medulla), and pCASL (*r* = 0.94, *P* < 0.001 in cortex and *r* = 0.58, *P* = 0.025 in medulla) (Fig. 5). No correlation was observed between RBF and eGFR for either approach (*r* ≤ 0.45, *P* > 0.05; data not shown).

**Table 2:** RBF and ATT values obtained with multi-delay FAIR and pCASL labeling approaches in the cortex and medulla averaged over all subjects at two different visits (mean ± SD)

	Visit 1^a^	Visit 2^b^	*P* value^c^	CV_ws_ [%]	ICC^d^ (95%-CI)
FAIR					
RBF (mL/min/100 g)					
Cortex	362 (57)	389 (55)	0.42	9.9	0.51 (− 0.058 to 0.83)
Medulla	140 (47)	135 (32)	0.34	13.8	0.80 (0.46–0.94)
ATT (s)					
Cortex	0.47 (0.13)	0.50 (0.13)	0.18	10.7	0.83 (0.53–0.95)
Medulla	0.70 (0.10)	0.71 (0.06)	0.23	8.1	0.40 (− 0.18 to 0.78)
pCASL					
RBF (mL/min/100 g)					
Cortex	201 (72)	207 (64)	0.57	33.9	− 0.38 (− 0.86 to 0.27)
Medulla	84 (27)	85 (41)	0.85	30.9	0.41 (− 0.23 to 0.79)
ATT (s)					
Cortex	0.71 (0.25)	0.68 (0.23)	0.79	19.4	0.60 (0.069–0.86)
Medulla	0.86 (0.12)	0.88 (0.12)	0.34	9.8	0.53 (− 0.008 to 0.83)

*ATT* arterial transit time, *CI* confidence interval, *CV*_*ws*_ within-subject coefficient of variation, *ICC* intra-class correlation coefficient, *RBF* renal blood flow, *SD* standard deviation

^a^Based on 15 subjects

^b^Based on 12 subjects

^c^Group differences between visits were tested with Wilcoxon matched-pair signed-rank test

^d^Two-way model, absolute agreement, single measures

**Fig. 6:**
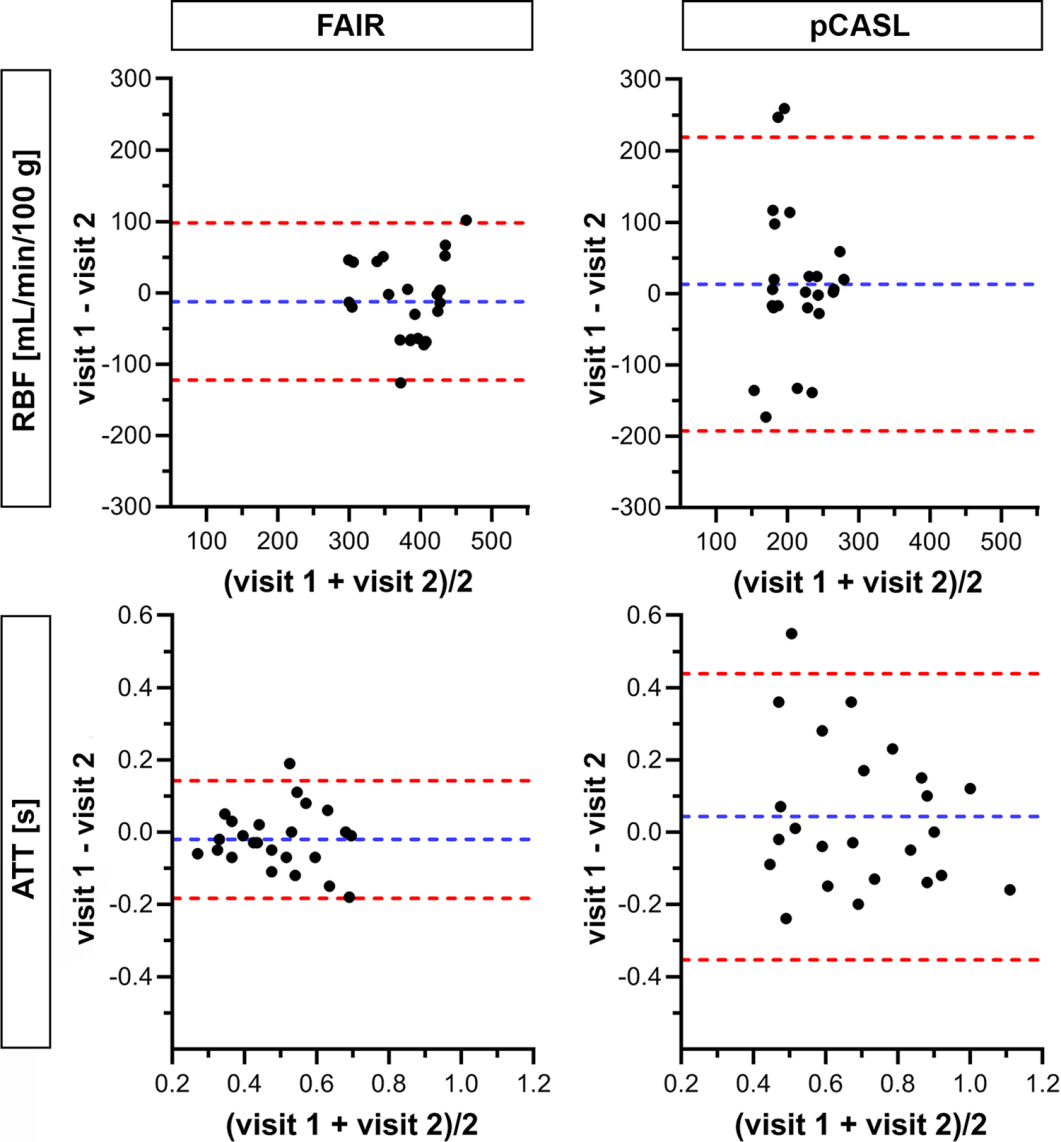
Bland–Altman plots comparing cortical RBF and ATT measurements between visits for both labeling approaches. Data points represent 24 kidneys from 12 subjects available at both visits. Blue and red dotted lines correspond to mean difference and limits of agreement, respectively. *ATT* arterial transit time, *RBF* renal blood flow

**Fig. 7:**
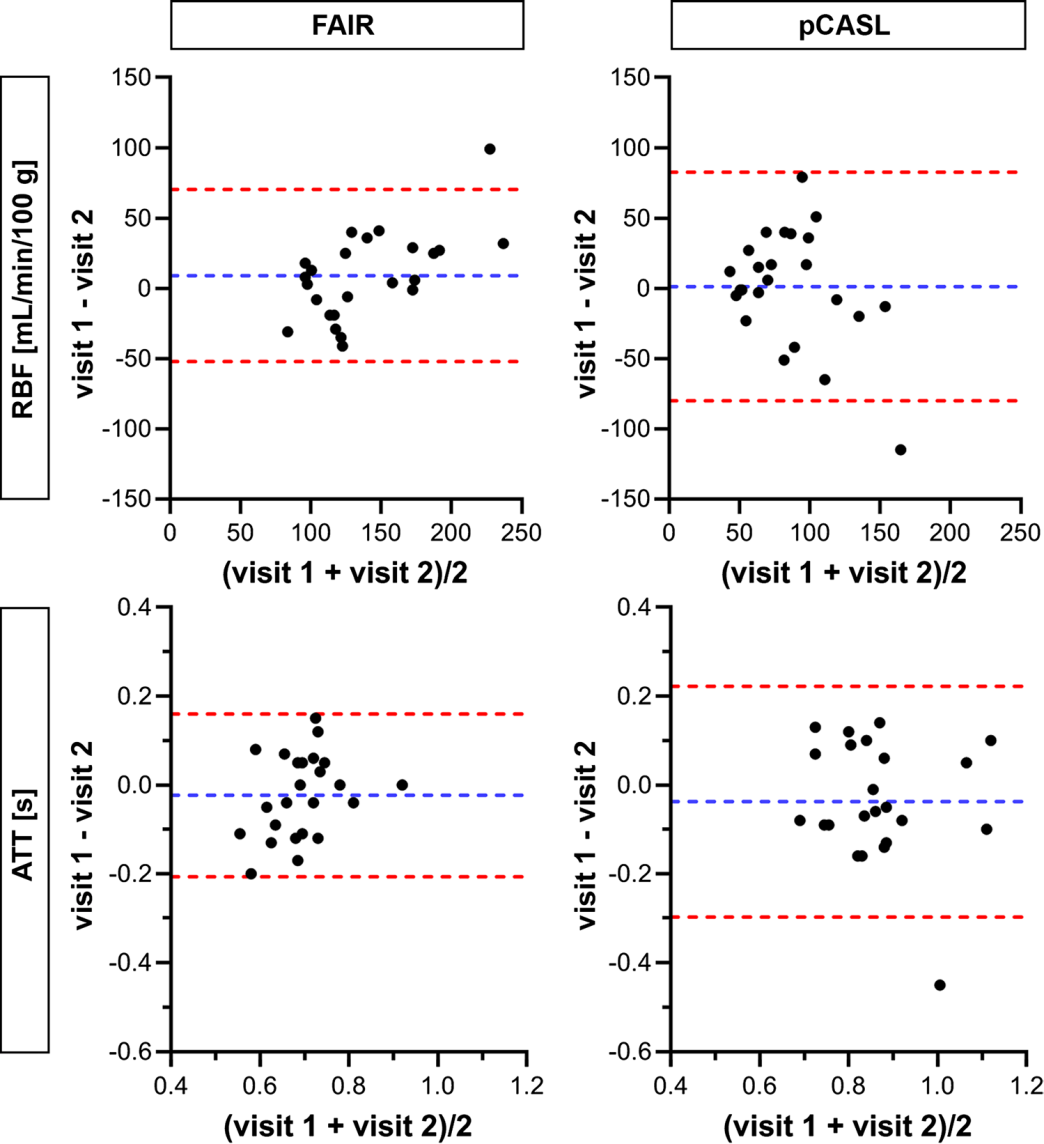
Bland–Altman plots comparing medullary RBF and ATT measurements between visits for both labeling approaches. Data points represent 24 kidneys from 12 subjects available at both visits. Blue and red dotted lines correspond to mean difference and limits of agreement, respectively. *ATT* arterial transit time, *RBF* renal blood flow

**Fig. 8:**
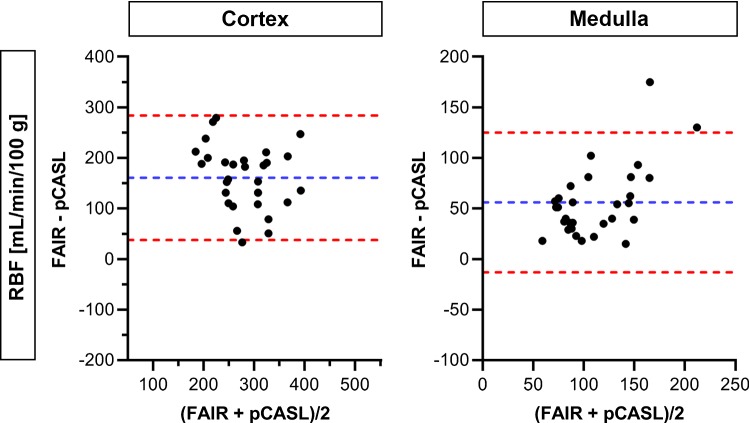
Bland–Altman plots comparing RBF measurements between labeling approaches for cortex and medulla. Data points represent 30 kidneys from 15 subjects at the first visit only. Blue and red dotted lines correspond to mean difference and limits of agreement, respectively. *RBF *renal blood flow

Bland–Altman plots comparing RBF measurements between visits for both labeling approaches are shown in Figs. 6 and 7 for cortex and medulla, respectively. A better inter-visit agreement was observed with FAIR in both the cortex and medulla. The ICC for FAIR was moderate for cortex and good for medulla, and the CV_ws_ was relatively low for both kidney regions. The ICC for pCASL was poor, and the CV_ws_ was high for both kidney regions (Table 2). On average, RBF values measured using FAIR were 161 mL/min/100 g higher for cortex and 56 mL/min/100 g higher for medulla, than when measured using pCASL (Fig. 8).

#### ATT

Mean cortical ATT was 0.47 ± 0.13 s with FAIR and 0.71 ± 0.25 s with pCASL at visit 1. Mean medullary ATT was 0.70 ± 0.10 s with FAIR and 0.86 ± 0.12 s with pCASL at visit 1. For both labeling approaches, ATT values were not significantly different between visits (*P* ≥ 0.18; Table 2), and neither between left and right kidneys of each subject for pCASL (*P* = 0.49 in cortex; *P* = 0.45 in medulla). However, for FAIR the ATT was significantly different between left and right kidneys of each subject (0.50 ± 0.13 and 0.45 ± 0.14 s, *P* = 0.010 in cortex for left and right kidneys, respectively; 0.74 ± 0.10 and 0.67 ± 0.12 s, *P* = 0.022 in medulla for left and right kidneys, respectively). There was a significant correlation between the measured ATT values in the left and right kidneys of each subject, both with FAIR (*r* = 0.86, *P* < 0.001 in cortex and *r* = 0.64, *P* = 0.011 in medulla), and pCASL (*r* = 0.95, *P* < 0.001 in cortex and *r* = 0.80, *P* < 0.001 in medulla) (Fig. 5).

Bland–Altman plots comparing ATT measurements between visits for both labeling approaches are shown in Figs. 6 and 7 for cortex and medulla, respectively. A better inter-visit agreement was observed with FAIR in the cortex, whilst agreement was comparable for both labeling approaches in the medulla. The ICC for FAIR was good for cortex and poor for medulla, and the CV_ws_ was relatively low for both kidney regions. The ICC for pCASL was moderate for both kidney regions, and the CV_ws_ was high for cortex and relatively low for medulla (Table 2).

## Discussion

The current study focused on comparing multi-delay ASL measurements with the two labeling approaches most commonly used in the kidney, FAIR and pCASL. The goal was to obtain a better insight into the reliability and repeatability of each method and to identify the most efficient method to perform multi-delay renal perfusion quantification at 3T. The study showed that measured renal perfusion values depend on the labeling approach. Perfusion values were significantly higher for FAIR than for pCASL, and showed substantially larger variability between visits for pCASL compared to FAIR.

Previous renal ASL studies at 3T in healthy volunteers have reported mean cortical RBF in the study population ranging from 199–399 mL/min/100 g and 138–296 mL/min/100 g for FAIR and pCASL, respectively [2]. Studies using multiple delay times reported mean values ranging from 151–309 ml/min/100 g and 117–215 mL/min/100 g for FAIR and pCASL, respectively [27]. These values highlight a wide range between studies that used the same labeling approach, as well as indicate a tendency of FAIR to measure higher RBF values, as found in our results. The wide variation of perfusion values makes it difficult to compare results between studies, even when the same labeling approach was used. Apart from differences in the used scanner hardware and image readout [28], choices of parameters, such as labeling efficiency, blood-tissue partition coefficient, used in the kinetic model for perfusion quantification largely influence the obtained values. In this study, RBF values obtained with pCASL may be underestimated due to a lower labeling efficiency then assumed in the quantification. For renal application, the pCASL labeling efficiency is most likely influenced by a combination of several factors which will be discussed in more detail below.

In this study at 3T, repeatability was better for FAIR compared to pCASL. For some pCASL data sets (3 out of 27), the averaged perfusion-weighted images showed very low signal corresponding to cortical RBF values < 100 mL/min/100 g. Compared with RBF values obtained at the other visit or with FAIR labeling in the same subject, it seems that these pCASL measurements failed. In the Supplementary Figures S3, S4 and Table S1, pCASL results are presented after exclusion of these data sets. Exclusion of these data sets made the repeatability of pCASL much more comparable with FAIR. After elimination, FAIR still gave RBF values that were on average 147 mL/min/100 g higher for cortex and 54 mL/min/100 g higher for medulla than when measured using pCASL.

Despite hydration instructions and planning both visits at the same time of the day, intra-subject variation of perfusion values between visits was observed for both labeling approaches. Physiological variation of blood flow in the aorta [29, 30] might play a role here in the amount of labeled blood that is created and delivered to the kidneys. Although there was intra-subject variability between visits, the diagnostic value of perfusion imaging, showing regional differences within or differences between kidneys, might still be unaffected. Moreover, studies comparing patients with impaired kidney function and healthy controls found relatively large perfusion differences of the average cortical RBF between both groups, ranging from 66 to 202 mL/min/100 g [31, 32].

The number of studies reporting reliability and repeatability of renal ASL is limited, especially at 3T [2]. Previous studies using FAIR at 3T in healthy volunteers [33, 34] have reported inter-visit ICCs of 0.80 and 0.85, and CVs of 9.2 and 9.3% for cortical RBF. To our knowledge, no studies have been published yet reporting inter-visit reliability and repeatability at 3T for pCASL. However, there is one study that has investigated intra-visit reliability and repeatability of pCASL at 3T [35] reporting an ICC of 0.93, and CV of 14.4% for cortical RBF. Reliability and repeatability of ATT measurements have been investigated even less. One study reported intra-visit an ICC of 0.32, and CV of 33.6% for cortical ATT with pCASL at 3T [35].

The ATT is dependent on the used measurement method, and was, therefore, not directly compared between both labeling approaches. Previous studies using multiple delay times reported average cortical ATT values of 0.11–0.30 s for FAIR and 0.96–1.23 s for pCASL [27]. The obtained ATT is very much determined by the timing of the (first) delay times. Based on preliminary measurements in healthy volunteers, we chose four equidistantly spaced delay times covering the perfusion signal over time both before and after the expected perfusion signal peak in the cortex for most subjects, to enable proper fitting of the perfusion model for quantification. The perfusion signal curves averaged over all subjects (Fig. 4) indicate that the delay times were correctly placed to capture the signal peak, for both labeling approaches. To improve the accuracy of the ATT estimation, more measurements with short delay times could be added. In general, accuracy of perfusion estimation increases with the number of sampled delay times [36]. However, for clinical applicability of renal ASL, total acquisition time is restricted, limiting the number of delay times (and label–control repetitions) that can be obtained.

Measuring medullary perfusion is challenging. The medulla is much less perfused compared to the cortex (only ~ 10% of blood flowing into the kidneys flows through the medulla [37]), and the transit time of labeled blood is much longer, which results in more T_1_ decay before entering the medulla and lower tSNR, as was observed in this study. It is thus not surprising that medullary perfusion showed lower repeatability than cortical perfusion. Thus far, not many studies have reported medullary RBF and ATT values, and mainly focused on measuring cortical perfusion.

In the current study, lower PWS and tSNR values were found for pCASL compared with FAIR. This finding is not in line with the notion that the inherent SNR is higher for pCASL than for PASL techniques such as FAIR. For the brain, this has been shown by theoretical modeling of the perfusion signal, and has been demonstrated experimentally [9, 38]. The higher intrinsic SNR of pCASL in the brain builds first on the longer temporal duration of the labeled bolus, which is proportional to a larger volume of labeled blood that is delivered to the tissue and second on the closer proximity of the labeling location and the imaging slab, which reduces T_1_ decay. The geometry of pCASL used in the kidney, with labeling taking place approximately 15 cm upstream of the tissue, reverses the labeling proximity argument to the advantage of FAIR. For FAIR, spatial coverage of the non-selective labeling slab is limited by the transmit RF coil, resulting in a smaller volume of labeled blood in brain. However, for kidneys, body coil transmission is used and kidneys are positioned nearly at isocenter, so this is expected to result in a very minor difference in the labeled blood volume compared to pCASL. In addition, FAIR has an essentially flow velocity independent inversion efficiency [38], which may be beneficial for application areas with a broad distribution of flow velocities and pulsatile flow.

As previously indicated, the poorer performance of pCASL might also be a result of reduced labeling efficiency caused by (a combination of) several factors. First, labeling efficiency is sensitive to B_0_ inhomogeneities present at the pCASL labeling location (due to the proximity of air in the lungs), especially for this study performed at 3T. The labeling efficiency of the balanced pCASL implementation used is more sensitive to B_0_ offsets than that of the unbalanced variant. However, separate B_0_ shimming at the labeling location during acquisition was performed to mitigate this effect. Still, B_0_ shimming at the labeling location may fail, thus resulting in compromised labeling efficiency. Second, the presence of B_1_ inhomogeneities may have resulted in lower B_1_ than expected at the labeling location. Finally, in contrast to FAIR, the inversion efficiency of pCASL is dependent on flow velocity [38]. In the current study, default implemented labeling settings were used for pCASL, which have been optimized for brain application. Blood flow characteristics such as maximum blood flow velocity and pulsatility of the descending aorta are typically different compared with those in the brain feeding arteries [39, 40]. At higher flow velocities, the adiabatic condition will be violated resulting in less optimal inversion of blood spins [38]. Optimization of pCASL labeling parameters for renal application has been shown to improve robustness to off-resonance effects and aortic flow pulsatility [41]. To improve repeatability of perfusion quantification and to detect failed measurements on a subject level, we think that it is recommended to measure the labeling efficiency at the labeling location, similar as has been proposed for the brain [42, 43]. This will permit to judge the technical validity of the ASL measurement and to correct for the labeling efficiency in the quantification.

This study has limitations. First, for the pCASL labeling approach, two variants have been proposed, balanced and unbalanced [18]. The balanced variant, which was used in this study, has been shown to be more sensitive to B_0_ offsets. Switching to unbalanced pCASL may improve robustness to off-resonance effects, as has been demonstrated for brain ASL [44] and preliminary data is available that shows this for the kidneys as well [45]. Nevertheless, unbalanced pCASL has its own disadvantages such as vulnerability to subtraction artifacts caused by eddy currents due to the usage of different gradient waveforms in label and control condition [18]. A more extensive direct comparison between both pCASL variants should be made in future studies to further optimize this labeling approach and improve robustness. Second, renal perfusion lacks a gold standard technique to enable validation of RBF values obtained with both labeling approaches. Alternative techniques for measurement of renal perfusion include the para-aminohippurate (PAH) clearance method [7] and PET imaging (using ^15^O-labeled water) [46]. PAH clearance measurement involves multiple blood and urine samples over a time course of several hours and provides only information on total perfusion of both kidneys combined. PET imaging involves the infusion of a radioactive tracer, but enables localized perfusion measurements. However, even in the absence of a gold standard, the clinical value of a perfusion measurement technique is determined by its capability to measure clinically relevant perfusion differences and changes. Finally, the acquisition order of FAIR and pCASL scans was not randomized. All FAIR scans with varying delay times were always performed before the pCASL scans, resulting in a difference of ~ 10 min between acquisition of both labeling approaches. This small time difference will probably not explain the difference found between the two methods.

In conclusion, in this comparative study between multi-delay FAIR and balanced pCASL for renal perfusion measurements at 3T in healthy middle-aged volunteers, FAIR showed favorable repeatability. To improve repeatability of perfusion quantification and assess the technical validity of an ASL measurement, addition of a labeling efficiency measurement is recommended, especially for the balanced pCASL variant used in this study.

## Electronic supplementary material

Below is the link to the electronic supplementary material.

Supplementary file1 (PDF 1064 kb)
