# Supplementary material for: Comparison of multi-delay FAIR and pCASL labeling approaches for renal perfusion quantification at 3T MRI
Source: MAGMA. 2019 Dec 6;33(1):81–94. doi: 10.1007/s10334-019-00806-7 (PMC7021666; doi:10.1007/s10334-019-00806-7)
Supplement: Supplementary file 1 — Supplementary file1 (PDF 1064 kb) [file 10334_2019_806_MOESM1_ESM.pdf]

Supplementary Material

Comparison of multi-delay FAIR and pCASL labeling approaches for renal perfusion quantification at 3T MRI

Magnetic Resonance Materials in Physics, Biology and Medicine

Anita A. Harteveld, PhD[1], Anneloes de Boer, MD[1], Suzanne L. Franklin, MSc[1,2], Tim Leiner, MD PhD[1], Marijn van Stralen, PhD[1], Clemens Bos, PhD[1]

[1]Department of Radiology, University Medical Center Utrecht, Utrecht University, Utrecht, The Netherlands
[2]C.J. Gorter Center for high field MRI, Department of Radiology, Leiden University Medical Center, Leiden, The Netherlands

E-mail address corresponding author: a.a.harteveld-2@umcutrecht.nl

Supplementary Figure S1. Schematic planning of the ASL scans for both labeling approach on the localizer scan in axial, coronal and sagittal orientations (left kidney and aorta region). The upper row shows positioning of the FAIR selective inversion slab (yellow) excluding the feeding arteries of the kidneys, QUIPSSII saturation slab (green) including the feeding arteries, and image readout with five slices (blue) planned coronal-oblique along the long axis of the kidneys. The bottom row shows positioning of the pCASL labeling slab (yellow) placed perpendicular to the descending aorta and image readout with seven slices (blue) with equal orientation as the FAIR scan.

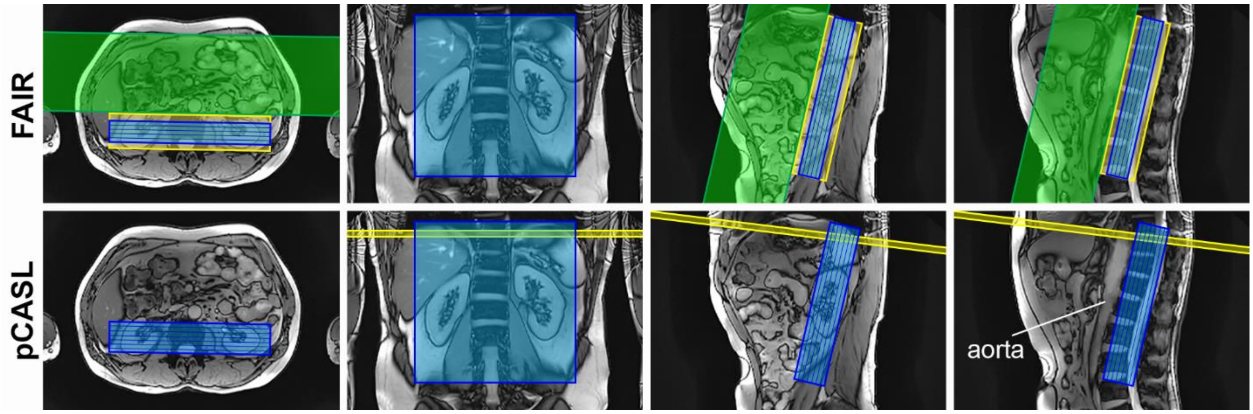

Supplementary Figure S2. Example of outlier rejection to exclude subtraction images. Images show masked subtraction images of the left kidney with FAIR labeling before outlier rejection. Subtraction images containing >20% voxels with a value of more than ±2SD from the mean voxel value over all repetitions were rejected. The label-control pairs (repetitions) that were removed after outlier rejection are highlighted in red. The color bar indicates PWS [%].

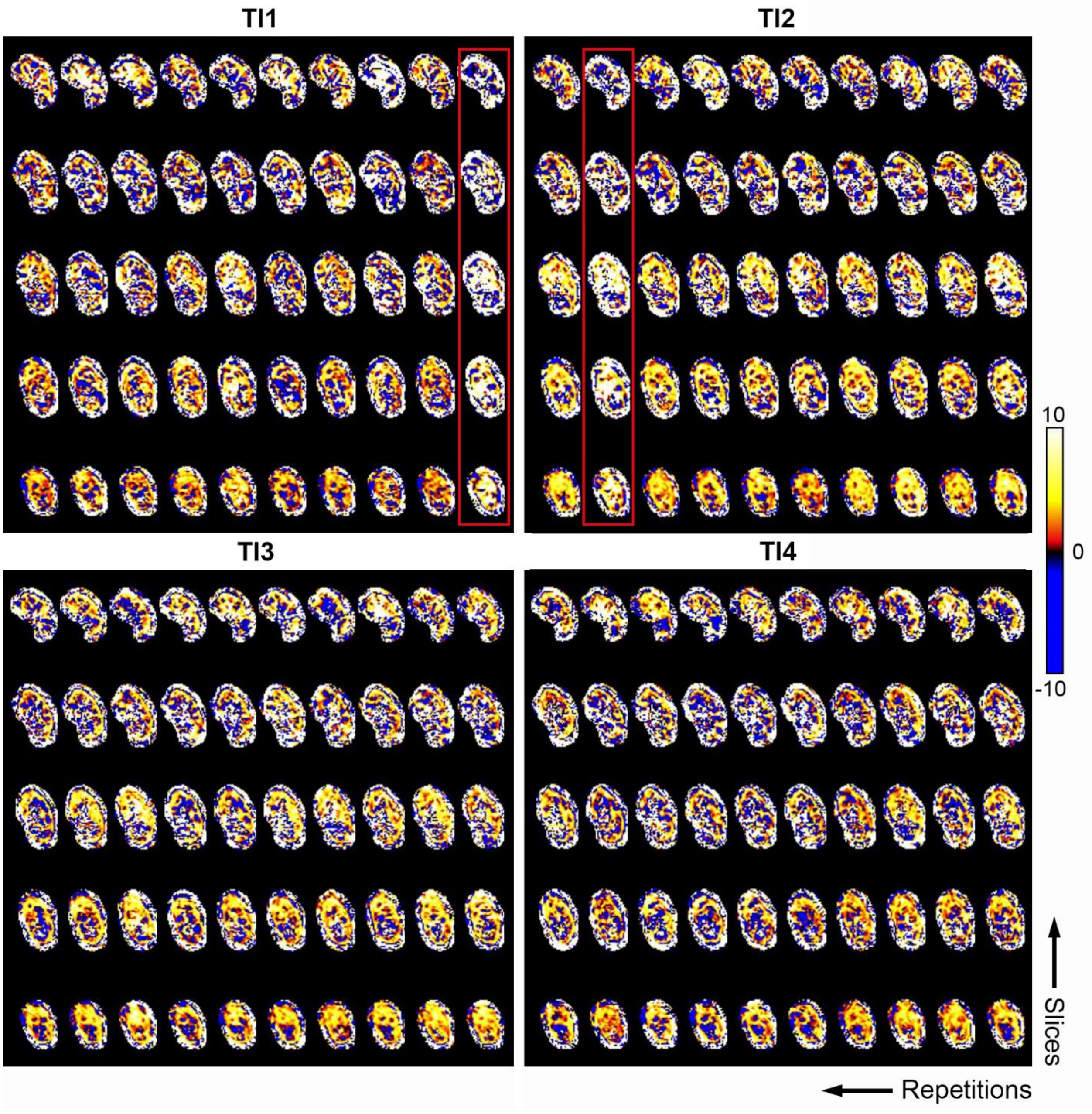

Supplementary Figure S3. Bland-Altman plots comparing RBF and ATT measurements between visits with pCASL in cortex and medulla after exclusion of datasets with cortical RBF values <100 mL/min/100 g. Data points represent 20 kidneys from 10 subjects available at both visits. Blue and red dotted lines correspond to mean difference and limits of agreement, respectively. *ATT: arterial transit time; RBF: renal blood flow.*

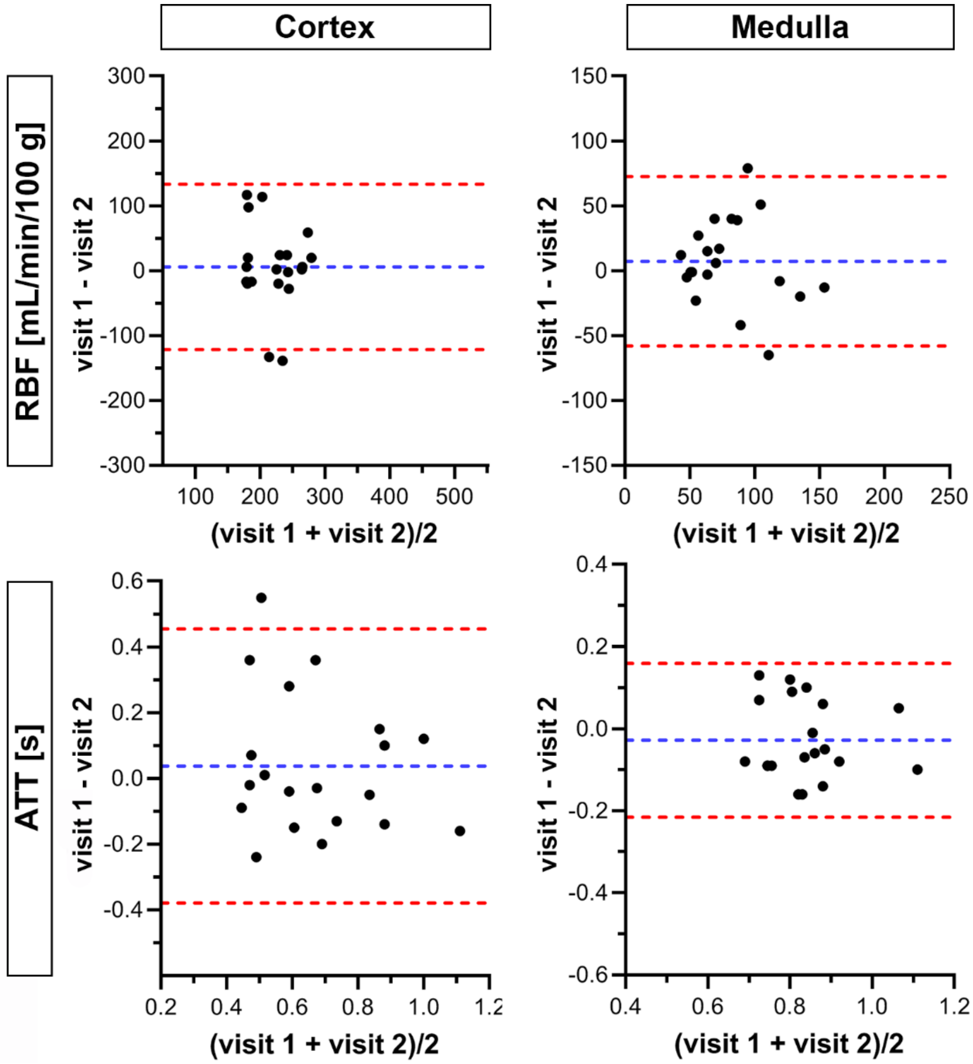

Supplementary Figure S4. Bland-Altman plots comparing RBF measurements between labeling approaches for cortex and medulla after exclusion of pCASL datasets with cortical RBF values <100 mL/min/100 g. Data points represent 26 kidneys from 13 subjects at the first visit only. Blue and red dotted lines correspond to mean difference and limits of agreement, respectively. *RBF: renal blood flow.*

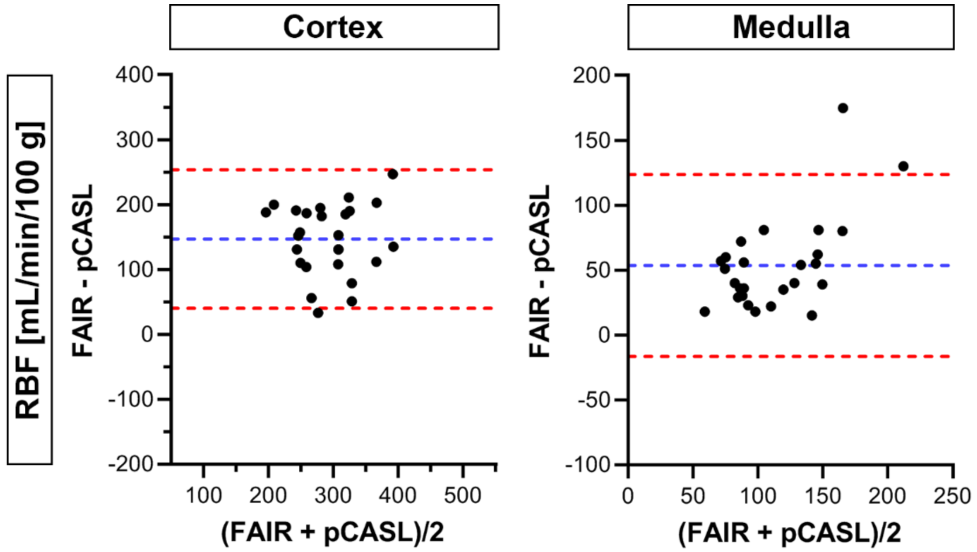

Supplementary Table S1. RBF and ATT values obtained with multi-delay pCASL labeling in the cortex and medulla averaged over all subjects at two different visits after exclusion of datasets with cortical RBF values <100 mL/min/100 g (mean±SD).

			**visit 1**[a]	**visit 2**[b]	***P*-value**[c]	**CV_{ws} [%]**	**ICC**[d] **(95%-CI)**
**pCASL**	RBF [mL/min/100 g]						
		cortex	220 (58)	219 (49)	0.49	19.7	0.10 (-0.63 – 0.68)
		medulla	86 (27)	85 (43)	0.70	26.6	0.57 (-0.046 – 0.87)
	ATT [s]						
		cortex	0.72 (0.26)	0.66 (0.23)	>0.99	21.0	0.59 (-0.035 – 0.88)
		medulla	0.84 (0.12)	0.86 (0.11)	0.28	7.1	0.71 (0.22 – 0.92)

[a]Based on 13 subjects.
[b]Based on 11 subjects.
[c]Group differences between visits were tested with Wilcoxon matched-pairs signed rank test.
[d]Two-way model, absolute agreement, single measures.
ATT: arterial transit time; CI: confidence interval; CV_{ws}: within-subject coefficient of variation; ICC: intra-class correlation coefficient; RBF: renal blood flow; SD: standard deviation.
